# A viral-vectored multipathogen vaccine platform based on replication-competent vaccinia virus and adeno-associated virus

**DOI:** 10.3389/fimmu.2026.1812029

**Published:** 2026-08-24

**Authors:** Yuna Sato, Yutaro Yamamoto, Ammar Abdurrahman Hasyim, Takeshi Morita, Koichi Watashi, Akihiko Sakamoto, Takuto Katayama, Kartika Hardianti Zainal, Mitsuhiro Iyori, Hiroaki Mizukami, Hisatoshi Shida, Shigeto Yoshida

**Affiliations:** 1 Laboratory of Vaccinology and Applied Immunology, Kanazawa University School of Pharmacy, Kanazawa, Ishikawa, Japan; 2 Department of Parasitology, Faculty of Medicine, Universitas Indonesia, Jakarta, Indonesia; 3 Department of Drug Development, National Institute of Infectious Diseases, Japan Institute for Health Security, Tokyo, Japan; 4 Research Institute of Pharmaceutical Sciences, Musashino University, Tokyo, Japan; 5 Division of Gene Therapy, Jichi Medical University, Shimotsuke, Tochigi, Japan; 6 Laboratory of Primate Model, Research Center for Infectious Diseases, Institute for Frontier Life and Medical Science, Kyoto University, Kyoto, Japan

**Keywords:** adeno-associated virus, LC16m8Δ, multipathogen vaccine, prime-boost immunization, vaccine platform, viral vector

## Abstract

Multi pathogen vaccines have emerged as a promising strategy to improve vaccine coverage, simplify immunization logistics, and address overlapping global health threats. However, few vaccine platforms have demonstrated robust and durable immune responses against pathogens spanning distinct biological classes. Here, we developed and evaluated a viral-vectored multipathogen vaccine platform based on a heterologous prime–boost regimen combining the highly attenuated vaccinia virus LC16m8Δ (m8Δ) and adeno-associated virus serotype 1 (AAV1). As a preclinical proof-of-concept, antigen-specific immune responses against *Plasmodium falciparum* and SARS-CoV-2 (Omicron variant) were evaluated, while neutralizing activity against mpox virus was assessed to examine the cross-reactive immunity conferred by the vaccinia virus vector. Immunogenicity, protective efficacy, and transmission-blocking activity were evaluated in murine models and mosquito feeding assays. The heterologous m8Δ prime/AAV1 boost regimen induced robust and long-lasting antigen-specific antibody responses that were maintained for up to 32 weeks. Complete sterile protection against transgenic *Plasmodium berghei* sporozoite challenge was achieved, together with greater than 90% transmission-blocking efficacy in mosquito feeding assays. In parallel, potent neutralizing antibody responses against SARS-CoV-2 Omicron and cross-neutralizing activity against mpox virus were observed. These findings demonstrate the feasibility of the m8Δ/AAV1 platform as a versatile viral-vectored multipathogen vaccine platform capable of integrating protozoan and viral antigens, providing a rationale for further preclinical optimization and clinical evaluation of next generation multipathogen vaccines targeting both endemic and emerging infectious diseases.

## Introduction

The dual burden of malaria and COVID-19 poses unprecedented challenges for sub-Saharan Africa, where already strained healthcare systems face overlapping epidemics and limited vaccine access (1, 2). Malaria caused by *Plasmodium falciparum* remains one of the leading causes of morbidity and mortality, particularly in sub-Saharan Africa, where children under five years of age are disproportionately affected (3, 4). Despite the introduction of the RTS, S/AS01, and R21 malaria vaccines, their moderate efficacy and waning durability highlight the need for improved platforms capable of providing more robust and sustained protection (5, 6).

The COVID-19 pandemic has added further complexity to this public health landscape (7, 8). In sub-Saharan Africa, SARS-CoV-2 vaccine coverage has lagged behind that of high-income countries due to supply shortages, cold-chain limitations, and distribution challenges. The emergence of immune-evasive variants such as Omicron has diminished the protective efficacy of existing vaccines, leaving these populations highly vulnerable (9, 10). In this context, developing a bivalent vaccine targeting both malaria and COVID-19 is of strategic importance, as it may reduce the logistical burden, improve vaccine uptake, and simultaneously address two major drivers of morbidity and mortality (11, 12).

The concept of multivalent and multipathogen vaccines has gained increasing attention as a means of improving immunological breadth and public health efficiency (13–16). Such platforms offer particular advantages in regions where the overlap of endemic infectious diseases and resource constraints amplifies the need for simplified immunization strategies (17). The vaccinia virus strain LC16m8Δ (m8Δ) is a genetically stable variant of LC16m8, which was used in smallpox vaccination of infants in Japan (18). It has a safety profile and the ability to induce robust immune responses (19–21). Given the close genetic relationship between the vaccinia virus and monkeypox virus (MPXV), neutralizing activity against MPXV elicited by strain LC16m8 is much stronger than the replication-incompetent modified vaccinia strain Anka (22). Additionally, the use of Adeno-associated virus serotype 1 (AAV1) as a booster vaccine vector strengthens and prolongs the immune response initially triggered by the m8Δ-prime vaccination (23). The capability of AAV1 to efficiently deliver genes and amplify the immune response is vital for ensuring long-term protection against rapidly evolving pathogens (23–25). We have recently shown that the LC16m8Δ-prime/AAV1-boost vaccine platform can elicit durable, long-lasting immune responses against both *P. falciparum* and *P. vivax* (26).

PfCSP and Pfs25 were selected as representative pre-erythrocytic and transmission-blocking malaria vaccine antigens, respectively, while Omicron S was selected as a representative SARS-CoV-2 antigen at the time the vaccine was designed. Together, these antigens represent pathogens of major public health importance and were used to evaluate the feasibility of the m8Δ/AAV1 platform as a multipathogen vaccination strategy. In the present study, we developed a trivalent viral-vectored vaccine, m8Δ/AAV1-Pf(s25-CSP)-Omicron S. By integrating *P. falciparum* and SARS-CoV-2 antigens within a single formulation, this approach could directly address the dual burden of malaria and COVID-19 in sub-Saharan Africa, while also extending cross-protection against mpox. The recent resurgence of mpox, including outbreaks in Africa and sporadic cases worldwide, underscores the urgency of vaccines capable of conferring durable immunity against this *Orthopoxvirus*. Beyond its immunological promise, this multipathogen vaccine platform carries translational significance by reducing logistical complexity, improving accessibility, and advancing disease elimination efforts in some of the world’s most vulnerable populations.

## Materials and methods

### Cells and viruses

Human embryonic kidney 293T (HEK293T) cells, baby hamster kidney cells, and rabbit kidney (RK13) cells were cultured as previously described (26). The African green monkey kidney-derived cell line (VeroE6 cells) and VeroE6 cells expressing human TMPRSS2 were cultured as detailed in a previous study (27). HEK293T cells co-expressing human ACE2 and TMPRSS2 have previously been described (28). SARS-CoV-2 Omicron strain TY38-873 was kindly provided by the National Institute of Infectious Diseases in Japan. Mpox strain Zr-599 (clade I) was propagated as previously described (29). All work involving SARS-CoV-2 and mpox viruses was performed under biosafety level 3 conditions. Recombinant AAV1 vectors were produced as previously described (25), while the propagation and titration of m8Δ followed previously established protocols (23).

### Parasites and animals

The transgenic *P. berghei* parasites expressing PfCSP (PfCSP-Tc/Pb) were utilized for the malaria challenge testing, and the transgenic *P. berghei* parasites expressing Pfs25DR3 (*Pb*Pfs25DR3) were employed in the malaria transmission-blocking assays, as previously described (23). These transgenic parasite strains were maintained in the Laboratory of Vaccinology and Applied Immunology at Kanazawa University. Six-week-old female ddY and BALB/c mice were purchased from Japan SLC (Hamamatsu, Shizuoka, Japan). Immune responses, protective efficacy, and transmission-blocking efficacy were assessed using BALB/c mice. *Anopheles stephensi mosquitoes* (SDA 500 strain) were used as hosts for transgenic parasites after feeding on the parasite-infected 6-week-old BALB/c mice. In the present study, female BALB/c mice obtained from Japan SLC, were consistently used at the age of 6–8 weeks.

### Viral vector construction

The recombinant vaccinia virus, m8Δ-Pf(s25-CSP)-HA, was created through homologous recombination for foreign gene insertion into the HA gene locus as previously described (23). The recombinant adeno-associated virus type-1, AAV1-Pf(s25-CSP), was also previously described (28).

For constructing the transfer vector for the AAV1 vaccine intended to deliver the SARS-CoV-2 Omicron S protein, a DNA fragment encoding amino acids of SARS-CoV-2 Omicron BA.1 spike protein (GISAID, accession no. EPI_ISL_6640919) was synthesized with mammalian codon optimization (GeneScript, Piscataway, NJ, USA). This fragment was then cloned into the *Eco* RI/*Age* I sites of pcDNA3.1-Wuhan-Hu-1 (28). The resulting plasmid, pcDNA3.1-Omicorn S, was subsequently digested with *Sac*I and *Xho* I and inserted into the *Sac* I/*Xho* I sites of pENTR-CMV-2020C3, which encodes the Wuhan-Hu-1 S protein with a deleted furin cleavage site. The modified plasmid, pENTR-CMV-Omicron S delFurin, was digested with *Not* I and *Asc* I, and the full-length gene encoding Omicron S delFurin protein under the control of the CMV *ie* promoter was inserted into the *Not* I/*Asc* I sites of pAAV-Asc I-MCS, which has an *Asc* I site in pAAV-MCS (backbone from (28); modified in this study). AAV1-Omicron S delFurin was then produced by transfecting HEK293T cells with the resultant plasmid, pAAV-CMV-Omicron S delFurin, as previously described (28).

To construct the transfer vector for the m8Δ vaccine, the *Age* I site (ACCGGT) of pENTR-CMV-Omicron S delFurin was altered to ACCGGC using the Q5 site-directed mutagenesis kit (New England Biolabs, Ipswich, MA, USA). The Omicron S delFurin fragment, which includes the *Age* I and *Pac* I digestion sites, was amplified using the primers pcDNA3.1-SARS-Omicron S and pENTER-CMV-2020C3 and subsequently cloned into a pMiniT vector (Gene script). The resultant plasmid, pMiniT-Omicron S delFurin-Age I, was digested with *Age* I and *Pac* I, and the full-length gene encoding Omicron S delFurin protein was inserted into the *Age* I/*Pac* I sites of pUC57-A46R-mPH5-p11-iRFP670 (28). The recombinant m8Δ-Pf(s25-CSP)-HA-(Omicron S delFurin-iRFP670)-A46R was generated by transfecting HEK293T cells, previously infected with m8Δ-Pf(s25-CSP)-HA, with the resultant plasmid pUC57-A46R-mPH5-Omicron S delFurin-p11-iRFP670. Single plaques expressing the iRFP670 fluorescent protein were selected and purified from RK cells using fluorescence microscopy, as previously described (26, 30).

### Virus transduction

HEK293T cells were infected with a recombinant m8Δ virus, specifically m8Δ-Pf(s25-CSP)-HA-Omicron S-iRFP670-A46R or m8Δ-Pf(s25-CSP), at a multiplicity of infection (MOI) of 5 for 48 h. Similarly, HEK293T cells were infected with either AAV1-Pf(s25-CSP) or AAV1-Omicron S at an MOI of 10^5.^ for 48 h. For western blot analysis, cell lysates were subjected to 10% SDS-PAGE, and the separated proteins were transferred onto an Immobilon FL^®^ PVDF membrane (Merck Millipore, Darmstadt, Germany). The membrane was then blocked using 5% skim milk in PBS. For detecting malaria antigens, the primary antibodies used were anti-PfCSP mAb (2A10) or anti-Pfs25 mAb (4B7), followed by detection with IRDye 800-conjugated goat anti-mouse IgG secondary antibody (Rockland Immunochemicals, Gilbertsville, PA, USA). For detection of Omicron S antigens, membranes were incubated with a primary antibody, anti-RBD mAb (SARS-CoV-2 Spike RBD, Omicron Variant; GeneScript, Nanjing, China), followed by a secondary antibody, IRDye 800CW-conjugated goat anti-mouse IgG (Rockland Immunochemicals). Protein bands were visualized using an Odyssey infrared imaging system (LI-COR Biosciences, Lincoln, NE, USA), as described previously (31).

### Immunofluorescence assays

RK cells were seeded at a density of 5 × 10^4^ cells per well and infected with the m8Δ vaccines by serial dilution. At 72 h post-infection, when viral plaques had formed, live-cell immunofluorescence staining was conducted. PfCSP was detected using R-phycoerythrin LK23-conjugated anti-PfCSP mAb 2A10, which had been directly labeled with the Alexa Fluor™ 594 Microscale Protein Labeling Kit (Invitrogen, Carlsbad, CA, USA). For detection of SARS-CoV-2 S1, an anti-SARS-CoV-2 S1 RBD human IgG1 mAb (clone 414-4; BioLegend, San Diego, CA, USA) was used as the primary antibody, followed by Alexa Fluor™ 488-conjugated goat anti-human IgG (H+L) secondary antibody (A18805; Invitrogen). Images of stained plaques were acquired using a BZ-X710 fluorescence microscope (Keyence Corp., Tokyo, Japan).

### Immunization

The m8Δ vaccines were administered via tail scarification at a dose of 1 × 10^7^ PFU as the prime immunization. The AAV1 vaccines were subsequently administered as a booster at a dose of 1 × 10^10^ viral genomes, with a 6-week interval. For the AAV1 vaccine combination regimen, equal doses of AAV1-Pf(s25-CSP) and AAV1-Omicron S were mixed in a single syringe for the booster. As a control, untreated (naive) mice were used. (**Supplementary Figure S1**).

### ELISA

PfCSP, Pfs25, and Omicron S antibody titers were quantified by ELISA as previously described (26, 32). The Pfs25 protein was produced using a wheat germ cell-free protein expression system (Cell-Free System, Matsuyama, Japan), as previously described (32). The commercial HEK293 HPLC-verified Omicron RBD protein (GenScript, Piscataway, New Jersey, USA #Z03728-100) was used. The 96-well microplates (Costar EIA/RIA polystyrene plates, Corning Inc., Corning, New York, NY, USA) were coated with 200–400 ng/ml of each antigen overnight at 4°C and then blocked with PBS containing 1% BSA. Tail blood samples were serially diluted, then added to the wells and incubated at room temperature for 1 h. After washing, HRP-conjugated goat anti-mouse IgG (H+L) antibodies (Bio-Rad, Tokyo, Japan) were added to the wells. A substrate solution for peroxidase containing H_2_O_2_ and 2,2^9^-and-bis(3-ethylbenzothiazoline-6-sulfonate) was used to develop the plates. The end-point titers were denoted as the inverse of the most diluted sample that yielded an optical density of 0.15 units at 414 nm, surpassing the optical density of the negative controls, which was below 0.1 unit. Before vaccination, all mice in the study were confirmed to be seronegative.

### ELISpot

The PfCSP-specific cellular immune response was measured by an ELISpot assay, as previously described (23) with minor modifications. Briefly, splenocytes were stimulated with an in-house synthesized PfCSP-derived peptide pool or individual peptides. The PfCSP-derived peptides included P1 (LNELNYDNAGTNLYN), P2 (NYDNAGTNLYNELEM), P3 (NYDNAGTNL), P4 (YLNKIQNSLSTEWSP), P5 (IQNSLSTEWSPCSVT) and P6 (PfCSP peptide pool). The PfCSP peptide pool consisted of 54 overlapping peptides (15-mers overlapping by 11 amino acids) spanning the sequence from LFQEYQCYGSSSNTR to KICKMEKCSSVL, which covers the central NANP/NVDP repeat and C-terminal regions of PfCSP.

For SARS-CoV-2-specific stimulation, splenocytes were incubated with spike-derived peptides, including P7 (GYLQPRTFL, aa 268–276) and P8 (KNKCVNFNF, aa 535–543), derived from the Omicron variant S protein. Non-stimulated splenocytes were included as negative controls.

IFN-γ ELISpot assays were performed using the BD™ IFN-γ ELISPOT Set (BD Biosciences, Franklin Lakes, NJ, USA), according to the manufacturer’s instructions. In brief, 96-well ELISpot plates were coated overnight at 4°C with anti-mouse IFN-γ capture antibody, washed, and blocked with complete RPMI 1640 medium for 2 h at 37°C. Splenocytes (1 × 10^6^ cells/well) were then added and stimulated with peptides (5 μg/ml each) for 18–20 h at 37°C in a 5% CO_2_ incubator. After incubation, plates were washed and incubated with biotinylated anti-IFN-γ detection antibody, followed by streptavidin–HRP and AEC substrate. Spots were visualized, air-dried, and counted using an ELISpot reader (Autoimmun Diagnostika, Strassberg, Germany). Data were expressed as spot-forming units per 10^6^ splenocytes after subtracting background counts from wells containing medium alone.

### Malaria challenge test

Mice were challenged with PfCSP/Pb sporozoites resuspended in RPMI 1640 medium. Sporozoites were prepared as previously described (33). Each mouse was administered 100 µl of media containing 1,000 sporozoites via the tail vein. The progression of malaria infection was monitored on days 4–14 by Giemsa staining of thin blood smears obtained from the tail. Protection was characterized by the absence of blood-stage parasitemia on day 14 post-challenge. The duration to achieve 1% parasitemia was determined as previously described (34). Protective efficacy was defined as the complete absence of detectable blood-stage parasites in peripheral blood smears until day 14, and a model predicting the time to reach 1% parasitemia was generated.

### Transmission-blocking assay

Transmission-blocking efficacy was assessed using direct-feeding assays as previously described (26). In brief, at 25 days post-boost, the mice were treated with phenyl hydrazine and infected intraperitoneally with 1 × 10^6^ *P. berghei* Pfs25DR3-parasitized RBCs 3 days later. At 3 days post-infection, at least 50 starved *Anopheles stephensi* mosquitoes were allowed to feed on each infected mouse. At 5–6 h post-feeding, any unfed mosquitoes were removed. On days 10–12 post-feeding, the midguts from the blood-fed mosquitoes were dissected, and the oocyst-positive percentage and intensity were recorded. Subsequent experiments were performed as described above. Percent (%) inhibition of the mean oocyst intensity (transmission-reducing activity; TRA) was calculated as follows: 100 × [1 - (mean number of oocysts in the test group/mean number of oocysts in the control group)]. The % inhibition of the oocyst-positive percentage (TBA) was evaluated as: 100 × [1 - (proportion of mosquitoes with any oocysts in the test group)/(proportion of mosquitoes with any oocysts in the control group)].

### Pseudovirus neutralization assay

Pseudovirus was produced and titrated as previously described (28). Briefly, cell culture dishes were coated with Cellmatrix type I-C collagen (Nitta Gelatin, Osaka, Japan). HEK293T cells were transduced with pcDNA3.1 encoding the S protein of SARS-CoV-2 Omicron stain TY38-873 (provided by the National Institute of Infectious Diseases in Japan) using effectene. One day later, the cells were inoculated for 2 h with the VSV backbone at the 50% tissue culture infectious dose (TCID_50_) of 8 × 10^4^ per 10^6^ cells and cultured overnight in fresh medium. Culture supernatants were then harvested as the pseudovirus and stored at -80°C until use. For the neutralization assay, the pseudovirus was incubated with serially diluted serum samples in triplicate for 1 h at 37°C. Freshly trypsinized HEK293T cells stably co-expressing human ACE2 and human TMPRSS2 were then mixed with the pseudovirus at the TCID_50_ of 3 × 10^2^ per 2 × 10^4^ cells and cultured overnight. Luciferase expression was measured on a GloMax 96 microplate luminometer (Promega, Madison, WI, USA) using the luciferase assay system (Promega). As a negative control, the G protein-pseudotyped recombinant VSV carrying the luciferase gene (Kerafast, Boston, MA, USA) was used.

### SARS-CoV-2 neutralization assay

The virus neutralization assay was performed in a biosafety level 3 laboratory as previously described (28). The BA.1 SARS-CoV-2 strain TY38-873 was incubated with serially diluted serum samples in triplicate for 1 h at 37°C. VeroE6 cells expressing human TMPRSS2 were then inoculated with this mixture at the TCID_50_ of 32 per 2 × 10^4^ cells and cultured for 3 days. Metabolically active cells were stained with 0.5 mg/ml 3-(4,5-dimethyl-2-thiazolyl)-2,5-diphenyltetrazolium bromide in serum-free medium, and lysed in isopropyl alcohol containing 4 mM HCl and 0.1% NP-40. Neutralizing activities were analyzed on a Multiskan GO microplate reader (Thermo Fisher Scientific, Waltham, MA, USA).

### MPXV neutralizing assay

For measuring the mpox neutralizing activity of immune mouse sera, the sera were serially diluted (beginning at 1:16) with DMEM containing 2% FBS mixed with an equal volume of MPXV Zr-599 (MOI=0.05) and incubated at 37°C for 24 h (35). VeroE6 cells seeded on 96-well plates were inoculated with 100 ml of the above virus-serum mixture for 24 h, followed by washing and the extraction of cellular DNA using a MagMAX DNA Ultra 2.0 with Cell and Tissue Extraction Buffer (Thermo Fisher Scientific) according to the manufacturer’s protocol. MPXV DNA was quantified by real-time PCR using TaqMan Gene Expression Master Mix (Thermo Fisher Scientific) with detection by QuantStudio 6 (Thermo Fisher Scientific). The primer set and a probe for detection of the F3L gene of MPXV (36) were as follows:

Forward: 5′-CATCTATTATAGCATCAGCATCAGA-3′

Reverse: 5′-GATACTCCTCCTCGTTGGTCTAC-3′

Probe: 5′-FAM-TGTAGGCCGTGTATCAGCATCCATT-MGB-3′

Neutralizing antibody titer (NT_50_) was defined as the dilution level of the serum that reduced 50% of viral DNA in cells compared with the control. The control serum used in this assay was derived from non-immunized mice.

### Cell viability assay

Cell viability was assessed using the Cell Counting Kit-8 (DOJINDO, Kumamoto, Japan) according to the manufacturer’s instructions. Cells in 96-well plates incubated with the diluted serum for 72 h were treated with 10 µl of Cell Counting Kit-8 reagent for 30 min, then the absorbance at 450 nm was measured using a microplate reader, Spark (TECAN, Zurich, Switzerland).

### Statistical analysis

Statistical analyses were conducted using GraphPad Prism version 9.0 for Mac OS. Differences between two groups were analyzed using the Mann–Whitney U test, and comparisons among multiple groups were assessed using the Kruskal–Wallis test followed by Dunn’s multiple comparisons. Prior to analysis, all ELISA end-point titers were transformed using a log10 scale. Antibody durability was assessed with Sidak’s multiple comparison test. The proportion of mice that did not reach 1% parasitemia was evaluated through a Kaplan–Meier log-rank (Mantel–Cox) test. Differences in TRA between groups were analyzed with a Mann–Whitney U test, while differences in TBA were examined using Fisher’s exact test. No formal *a priori* power calculation was performed. Sample sizes were determined based on our laboratory’s previous experience with this vaccine platform and established practice in preclinical vaccine studies.

## Results

### Construction and expression of the trivalent vaccine

**Figure 1:**
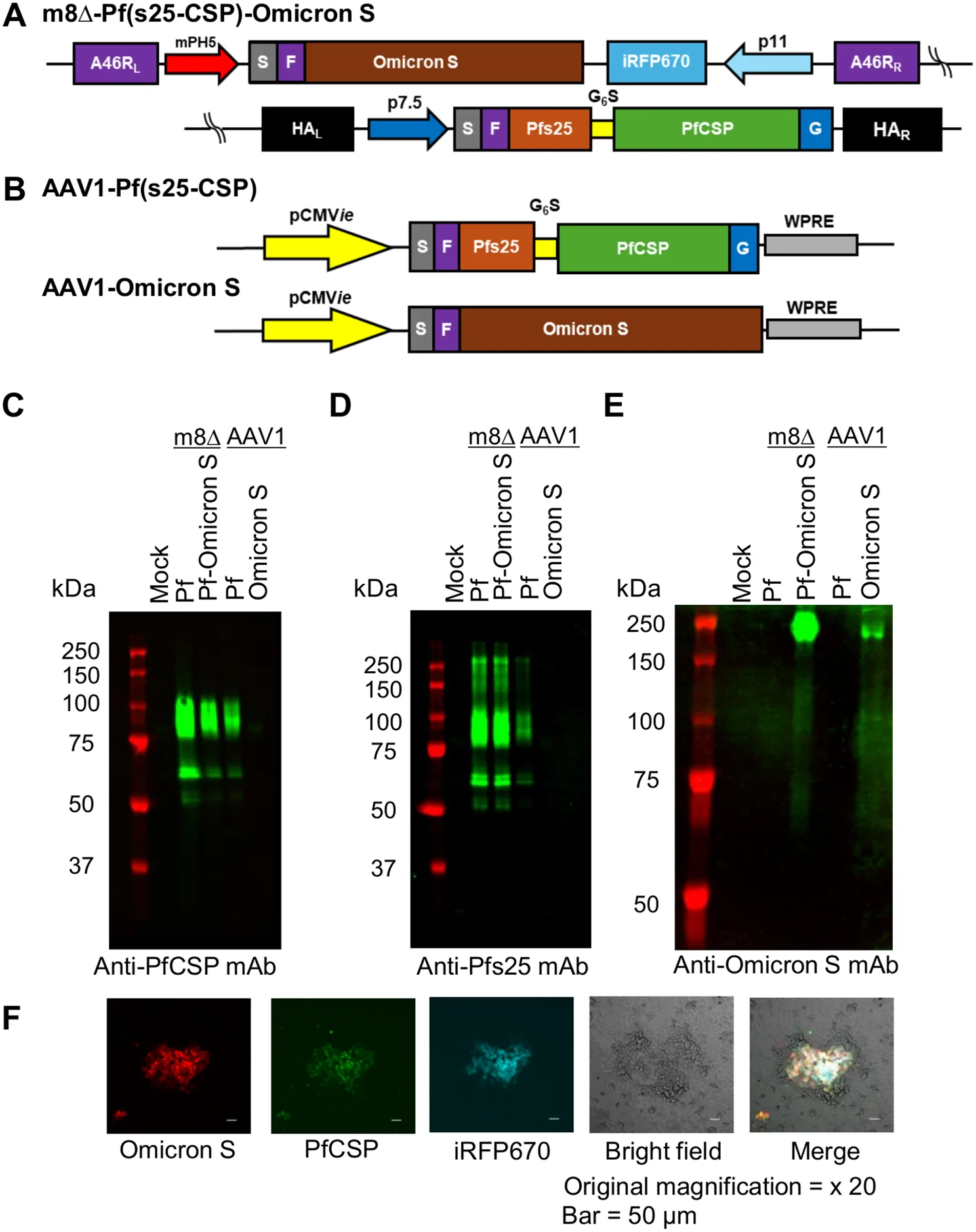
Vaccine design and antigen expression. **(A)** Schematic representation of the modular antigen cassette design, illustrating fusion. constructs that enable the incorporation of antigens derived from distinct pathogens into a single platform. **(B)** Overview of the heterologous prime-boost strategy combining LC16m 8Δ (m8Δ) vaccinia virus and adeno-associated virus (AAV), highlighting the flexibility of antigen insertion and promoter usage across vectors. **(C–E)** Immunoblot analysis confirming antigen expression from both viral vectors, demonstrating platform compatibility with diverse antigen constructs. For reference, the theoretical molecular weights of PfCSP, Pfs25, the Pf(s25-CSP) fusion protein, and Omicron S are 42 kDa, 25 kDa, 67 kDa, and 142 kDa, respectively. **(F)** Immunofluorescence analyses showing cell-surface and intracellular expression of platform-encoded antigens following transduction with AAV or infection with m8Δ. Together, these data illustrate the modularity, versatility of the LC16m8Δ/AAV platform, supporting its application to multipathogen and multistage vaccine development.

We generated a trivalent m8Δ-based vaccine encoding the *P. falciparum* Pf(s25-CSP) fusion protein and the SARS-CoV-2 Omicron spike (Omicron S) protein. The Pf(s25-CSP) gene cassette was inserted into the HA locus and the codon-optimized Omicron spike gene, with furin cleavage site deletion and stabilizing proline substitutions (K986P, V987P) (37), was inserted into the A46R locus, under the control of the P7.5 and mPH5 promoters, respectively. In parallel, AAV1-based vectors encoding either Pf(s25-CSP) or Omicron S under the CMV promoter were constructed (23, 24) (**Figures 1A, B**).

Western blotting of transduced HEK293T cells confirmed expression of the Pf(s25-CSP) fusion protein (~80–100 kDa), detected with mAbs 2A10 and 4B7, and Omicron S, detected with an anti-receptor-binding domain (RBD) antibody (**Figures 1C–E**). The Pf(s25-CSP) fusion protein migrated at a higher apparent molecular weight than its predicted size (~67 kDa), consistent with previous reports on PfCSP-containing proteins. Minor lower- and higher-molecular-weight bands likely represent degraded and aggregated forms, respectively. Omicron S migrated at a higher apparent molecular weight than its predicted size, consistent with the known extensive glycosylation of the SARS-CoV-2 spike protein (38). Comparable expression patterns were observed with AAV1-Pf(s25-CSP) and AAV1-Omicron S (**Figures 1C–E**, lanes 5 and 6). Furthermore, immunofluorescence analysis of viral plaques detected both Pf(s25-CSP) and Omicron S antigens, confirming the expression of the recombinant vector (**Figure 1F**).

### Potent and durable antibody responses

**Figure 2:**
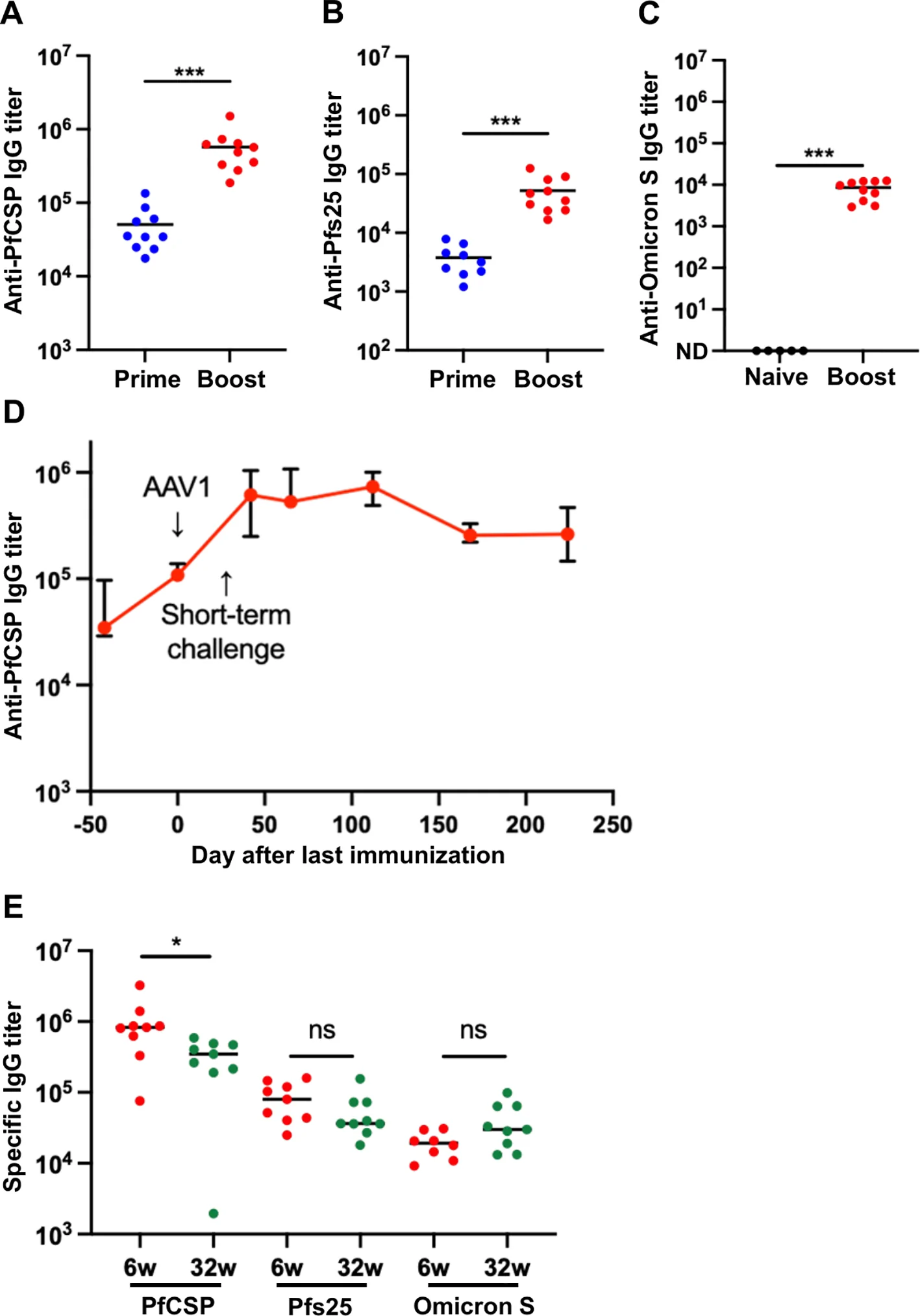
Induction of a durable antibody response. **(A–C)** BALB/c mice (n = 10) were immunized and sera collected before boost (Prime) and 4 weeks after boost (Boost); naive sera served as controls. Antibody titers against PfCSP **(A)**, Pfs25 **(B)**, and Omicron S **(C)** are shown. Each point represents an individual mouse, and the horizontal line indicates the mean. Mann-Whitney U test: ***p < 0.001; ND: not detectable. **(D)** Longitudinal anti-PfCSP antibody titers over 224 days post-immunization. Points represent the mean antibody titers, and error bars represent SD. **(E)** Comparison of anti-PfCSP, anti-Pfs25, and anti-Omicron antibody titer 6w (42days) and 32w (224 days) after the final immunization. Each point represents an individual mouse, and the horizontal line indicates the mean. *p < 0.05; ns, not significant.

Next, we assessed the humoral immune responses after a heterologous prime-boost regimen. BALB/c mice primed with m8Δ-Pf(s25-CSP)-Omicron S and boosted with AAV1- Pf(s25-CSP) plus AAV1-Omicron S at a 6-week interval (**Supplementary Figure S1A**) higher titers than the Prime group (*p* < 0.001). In addition, Omicron S-specific IgG titers were higher than those in the naïve group (*p* < 0.001). High titers were sustained for 32 weeks (Pfs25, 3.6 × 10^4^; PfCSP, 4.0 × 10^5^; Omicron S, 3.3 × 10^4^) (**Figure 2**).

### Cellular immune responses

**Figure 3:**
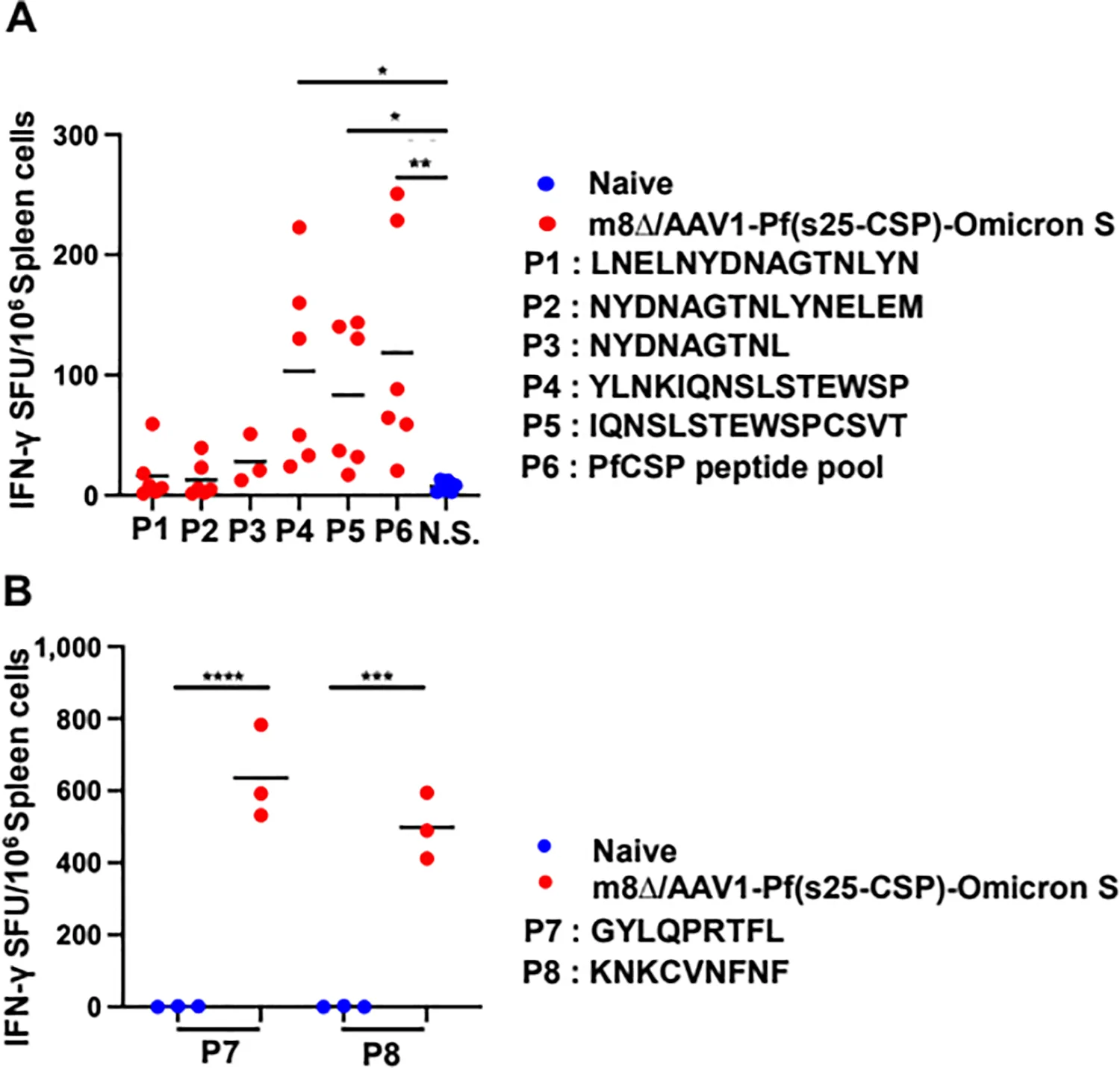
Cellular immune responses induced by the trivalent vaccine. **(A, B)** Splenocytes from immunized mice were stimulated with PfCSP peptides (overlapping pool and CD4+/CD8+ epitopes) or SARS-CoV-2 Omicron S peptides (#268, #535). IFN-γ- secreting cells were quantified by ELISpot. Statistical significance was assessed by Kruskal-Wallis with Dunn's multiple comparisons. ****p <0.0001; ***p < 0.001; **p < 0.01; *p <0.05; ns, not significant. N.S .; non-stimulated splenocytes.

Cellular immune responses elicited by the m8Δ/AAV1-Pf(s25-CSP)-Omicron S vaccine were evaluated by IFN-γ ELISPOT using splenocytes from immunized mice. To match the peptide numbering shown in **Figure 3** (P1–P8), splenocytes were stimulated with PfCSP-derived peptides (P1-P6) and SARS-CoV-2 Omicron S-derived peptides (P7-P8), as detailed in the Materials and Methods.

Significant antigen-specific IFN-γ responses were detected in splenocytes stimulated with PfCSP epitopes P4–P5 and P6 (*p* < 0.05 for P4–P5 and *p* < 0.01 for P6, respectively). In addition, immunized mice exhibited significantly higher frequencies of IFN-γ–secreting cells in response to P7–P8 compared with vector controls (*p* < 0.0001 and *p* < 0.001, respectively), demonstrating the induction of broad cellular immunity targeting both SARS-CoV-2 and *P. falciparum* antigens (**Figures 3A, B**).

### Protective efficacy against transgenic *P. berghei* lines expressing PfCSP

**Figure 4:**
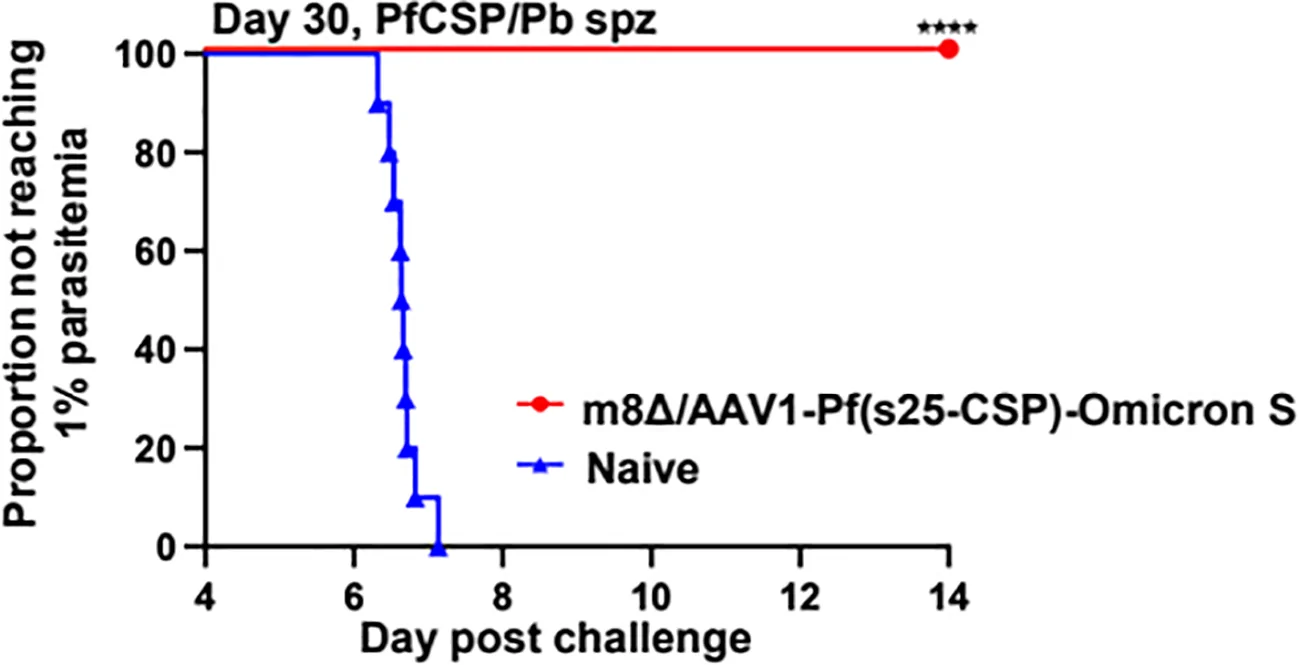
Protective efficacy against malaria challenge. At 30 days post-immunization, mice received either m8Δ/AAV1 (n = 10) or naïve (untreated) mice (n = 10), followed by challenge with PfCSP-Tc/Pb sporozoites. BALB/c mice were immunized with the m8Δ/AAV1-Pf(s25-CSP)-Omicron S vaccine. Survival curves were analyzed using the log-rank (Mantel-Cox) test, comparing each group to the naive control group. ****p <0.0001.

Protective efficacy of the vaccine was further assessed using transgenic *Plasmodium berghei* parasites expressing PfCSP. In challenge studies (day 30 post-immunization), all immunized mice (10/10, 100%, *p* < 0.0001) were completely protected, whereas all control animals developed parasitemia (**Figure 4**).

### Transmission-blocking efficacy

**Figure 5:**
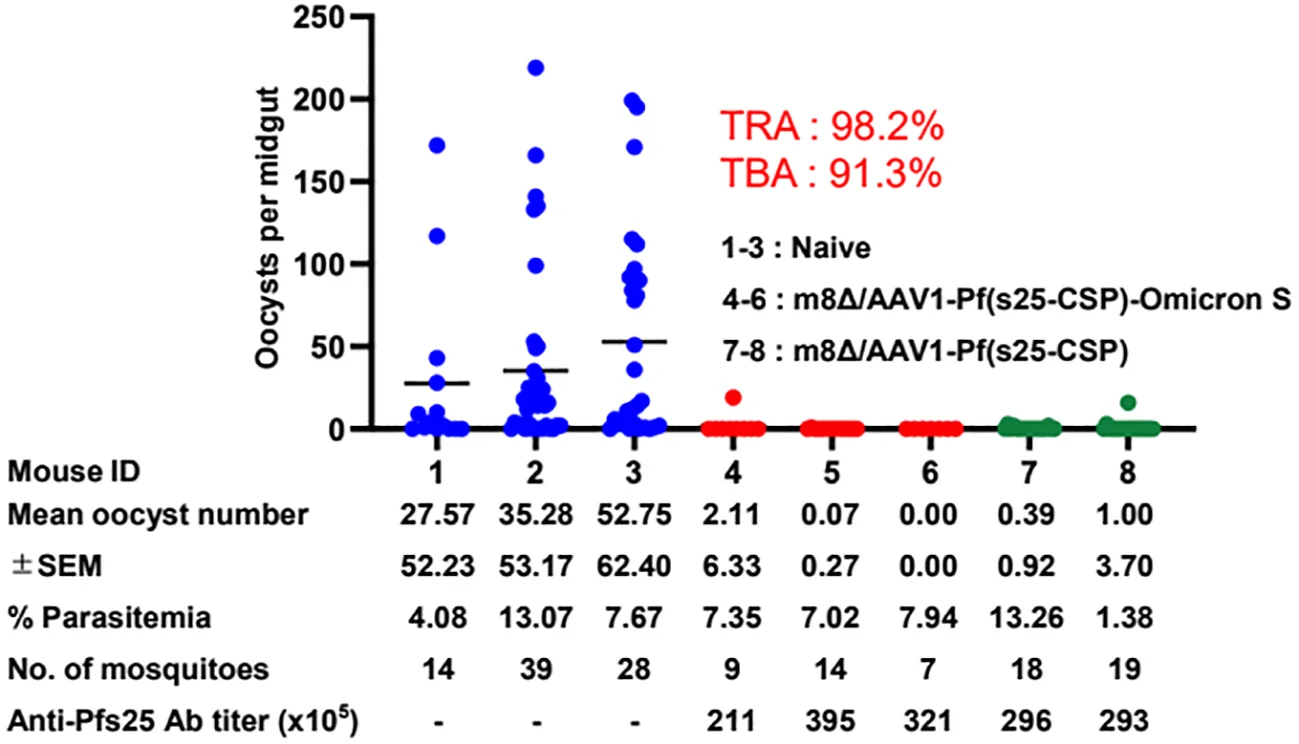
Transmission-blocking activity. Evaluation of transmission-blocking efficacy at 31 days (naive, n = 3; m8Δ/AAV1-Pf(s25-CSP)-Omicron S, n = 3; m 8Δ/AAV1-Pf(s25-CSP), n = 2) after the final immunization with m8Δ/AAV1-Pf(s25-CSP)-Omicron S. Each data point represents the number of oocysts in a single blood-fed Anopheles mosquito, with horizontal lines indicating mean oocyst counts. Statistical analysis was performed using the Mann- Whitney U test for TRA and Fisher's exact test for TBA compared with the control group. ****p < 0.0001; ns, not significant.

Transmission-blocking efficacy of the m8Δ/AAV1-Pf(s25-CSP)-Omicron S vaccine was evaluated by direct-feeding assays at 31 days after the final immunization (**Supplementary Figure S1C**). Immunized mice showed potent transmission-reducing activity (39) of 98.2% and transmission-blocking activity (TBA) of 91.3% compared with controls (*p* < 0.0001), with mean oocyst intensity reduced from 38.5 to 0.73 per midgut and the proportion of infected mosquitoes reduced from 77.8% to 10.9% (**Figure 5**).

These results confirmed that the vaccine confers potent transmission-blocking efficacy through significant reductions in both infection intensity and mosquito infection rate.

### Neutralizing activity against SARS-CoV-2 Omicron and MPXV

**Figure 6:**
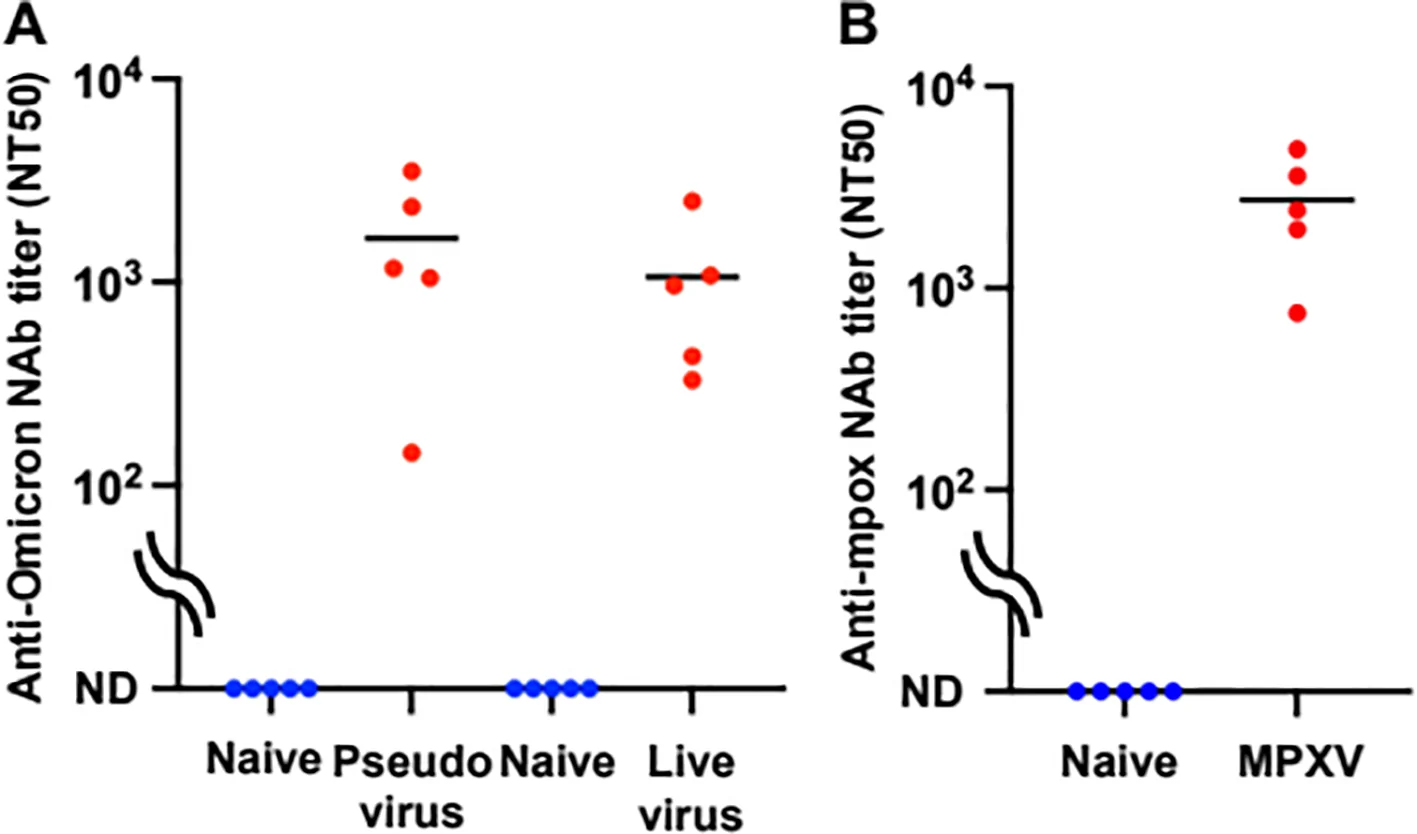
Neutralizing activity against SARS-CoV-2 Omicron and MPXV. **(A)** Neutralizing antibody titers (NT_50_) of sera collected four weeks after the booster dose of m8Δ-Pf(s25-CSP)-Omicron S were measured using both SARS-CoV-2 Omicron BA.1 pseudovirus and live virus. Each dot represents an individual mouse, and the horizontal line indicates the mean. ND: not detectable (below the limit of detection). **(B)** Neutralizing antibody responses against mpox virus (MPXV) were evaluated using sera collected four weeks after the booster immunization with m8Δ-Pf(s25-CSP)-Omicrons Neutralizing activity against the MPXV Zr-599 strain (clade I) was determined by quantifying the reduction in viral genome copies in infected Vero cells using real-time PCR. Each dot represents an individual mouse, and the horizontal line indicates the mean. ND: not detectable (below the limit of detection).

The neutralizing activity of sera from mice immunized with the m8Δ-Pf(s25-CSP)-Omicron S vaccine was evaluated against SARS-CoV-2 Omicron BA.1 pseudovirus and live virus, as well as mpox virus (MPXV). Serum samples collected four weeks post-boost demonstrated robust neutralization of the BA.1 pseudovirus, with the 50% neutralizing titer (NT_50_) values ranging from 900 to 4,000, and potent neutralization of live virus, with NT_50_ values of approximately 1,000 (**Figure 6A**). No neutralizing activity was detected in sera from control mice. Sera from immunized mice also significantly reduced MPXV DNA in infected Vero E6 cells in a dose-dependent manner. This neutralizing activity is consistent with the protective immunity previously demonstrated in LC16m8-immunized monkeys, which were completely protected against MPXV challenge (40) (**Figure 6B**, **Supplementary Figure 2**).

These results indicated that the m8Δ-Pf(s25-CSP)-Omicron S vaccine induces strong and neutralizing antibody responses against both SARS-CoV-2 Omicron and MPXV.

## Discussion

This study provides preclinical proof-of-concept for the feasibility of a heterologous viral-vectored multipathogen vaccine platform based on the highly attenuated vaccinia virus LC16m8Δ (m8Δ) and AAV1 in a prime–boost regimen. Using this platform, we simultaneously delivered antigens derived from *P. falciparum* and SARS-CoV-2 while also evaluating cross-neutralizing activity against mpox virus mediated by the vaccinia virus vector. This is the first study to demonstrate that a heterologous vaccinia virus/AAV platform can integrate protozoan and viral antigens within a single vaccine platform while inducing functional immune responses against biologically distinct pathogens.

A central advantage of this approach lies in the ability of heterologous viral-vector immunization to promote coordinated humoral and antigen-specific cellular immune responses. The m8Δ-prime/AAV1-boost regimen elicited robust antigen-specific antibody responses against PfCSP, Pfs25, and Omicron S that were maintained for at least 32 weeks. In addition, antigen-specific IFN-γ responses were detected following stimulation with malaria- and SARS-CoV-2-derived peptides, demonstrating the induction of antigen-specific cellular immune response against both target pathogens. Consistent with these immune responses, complete sterile protection against transgenic *P. berghei* sporozoite challenge was achieved. The vaccine also induced potent transmission-blocking activity in mosquito feeding assays. Furthermore, neutralizing antibody responses against SARS-CoV-2 Omicron together with cross-neutralizing activity against mpox virus were observed, highlighting an additional advantage of the vaccinia virus vector beyond its role as a vaccine delivery vehicle.

An important objective of this study was not simply to combine multiple antigens but to evaluate whether a single heterologous viral-vectored platform could accommodate pathogens with distinct biological characteristics. Malaria parasites require both protective immunity against liver-stage infection and transmission-blocking immunity, whereas SARS-CoV-2 primarily requires virus-neutralizing antibodies. The ability of the present platform to induce immune responses corresponding to these different protective mechanisms supports the feasibility of applying this platform to the development of multipathogen vaccines targeting pathogens of major public health importance.

Combination vaccines inevitably raise concerns regarding antigen competition and immune interference. Although these phenomena were not directly evaluated by direct comparison with corresponding monovalent vaccine formulations in the present study, the multivalent vaccine maintained complete protective efficacy against malaria challenge while simultaneously inducing robust antibody responses against SARS-CoV-2 and mpox virus. These findings suggest that simultaneous antigen delivery is feasible within this platform. Nevertheless, dedicated studies directly comparing multivalent and corresponding monovalent vaccine formulations will be required to determine whether immune interference occurs and to define its impact on the immunogenicity of each vaccine component. Addressing this question will be particularly important for future clinical evaluation of this platform.

The present study also highlights several practical considerations for clinical translation. Viral-vectored vaccines offer several important advantages (26), including potent immunogenicity, durable immune responses, and flexibility in antigen design. Conversely, important challenges remain, including pre-existing immunity to viral vectors, regulatory considerations associated with live viral vaccines, and manufacturing complexity (41). In particular, two independent AAV1 vectors were required because the combined transgene sequences exceeded the packaging capacity of a single AAV vector. Although this approach enabled simultaneous delivery of all target antigens, it inevitably increases manufacturing complexity and production costs. Future optimization of vector design or alternative delivery strategies may enable incorporation of all antigens into a single vector.

The heterologous m8Δ/AAV1 regimen used in the present study was selected based on our previous study, in which the AAV1 boost significantly enhanced antigen-specific antibody responses and sterile protection against malaria compared with m8Δ alone or homologous m8Δ vaccination (42). Therefore, the primary objective of the present study was not to re-evaluate the booster effect of AAV1 itself, but to determine whether this established heterologous platform could be extended to a multipathogen vaccine incorporating both protozoan and viral antigens. Nevertheless, because the present study did not include corresponding monovalent comparator groups under the new multipathogen vaccine configuration, the individual contribution of the AAV1 boost and the possibility of immune interference cannot be distinguished directly from the current data. Future studies incorporating appropriate monovalent comparator vaccine formulations will therefore be valuable for further optimizing the platform and defining the respective contributions of the prime and boost components.

Several limitations of this study should also be acknowledged. First, all efficacy studies were performed in murine models, and the extent to which these findings will translate to genetically diverse human populations remains uncertain. Second, Omicron S was selected because it represented the predominant circulating SARS-CoV-2 variant at the time the vaccine constructs were designed; therefore, protection against subsequently emerging variants was not evaluated. Finally, additional studies under clinically relevant conditions, including non-human primate studies and clinical studies, will be necessary before the translational potential of this platform can be adequately evaluated.

In conclusion, this study provides preclinical evidence supporting the feasibility of a heterologous m8Δ/AAV1 viral-vectored platform as a multipathogen vaccine platform. By integrating antigens from protozoan and viral pathogens within a single prime–boost regimen, this platform induced long lasting humoral immunity, antigen-specific cellular immune responses, sterile protection against malaria challenge, potent transmission-blocking activity, and neutralizing activity against SARS-CoV-2 and mpox virus. These findings provide a rationale for further preclinical optimization and eventual clinical evaluation of this platform as a flexible vaccination platform targeting pathogens of major public health importance.

## Data Availability

The original contributions presented in the study are included in the article/**Supplementary Material**. Further inquiries can be directed to the corresponding author.
